# Examining the Threat of ChatGPT to the Validity of Short Answer Assessments in an Undergraduate Medical Program

**DOI:** 10.1177/23821205231204178

**Published:** 2023-09-28

**Authors:** Leo Morjaria, Levi Burns, Keyna Bracken, Quang N. Ngo, Mark Lee, Anthony J. Levinson, John Smith, Penelope Thompson, Matthew Sibbald

**Affiliations:** 1 12362 Michael G. DeGroote School of Medicine, McMaster University, Hamilton, Ontario, Canada; 2 McMaster Education Research, Innovation and Theory (MERIT) Program, 3710 McMaster University, Hamilton, Ontario, Canada

**Keywords:** undergraduate, medical student, pre-clerkship, ChatGPT, artificial intelligence, short answer assessment

## Abstract

**OBJECTIVES:**

ChatGPT is an artificial intelligence model that can interpret free-text prompts and return detailed, human-like responses across a wide domain of subjects. This study evaluated the extent of the threat posed by ChatGPT to the validity of short-answer assessment problems used to examine pre-clerkship medical students in our undergraduate medical education program.

**METHODS:**

Forty problems used in prior student assessments were retrieved and stratified by levels of Bloom's Taxonomy. Thirty of these problems were submitted to ChatGPT-3.5. For the remaining 10 problems, we retrieved past minimally passing student responses. Six tutors graded each of the 40 responses. Comparison of performance between student-generated and ChatGPT-generated answers aggregated as a whole and grouped by Bloom's levels of cognitive reasoning, was done using t-tests, ANOVA, Cronbach's alpha, and Cohen's d. Scores for ChatGPT-generated responses were also compared to historical class average performance.

**RESULTS:**

ChatGPT-generated responses received a mean score of 3.29 out of 5 (n = 30, 95% CI 2.93-3.65) compared to 2.38 for a group of students meeting minimum passing marks (n = 10, 95% CI 1.94-2.82), representing higher performance (*P* = .008, η^2^ = 0.169), but was outperformed by historical class average scores on the same 30 problems (mean 3.67, *P* = .018) when including all past responses regardless of student performance level. There was no statistically significant trend in performance across domains of Bloom's Taxonomy.

**CONCLUSION:**

While ChatGPT was able to pass short answer assessment problems spanning the pre-clerkship curriculum, it outperformed only underperforming students. We remark that tutors in several cases were convinced that ChatGPT-produced responses were produced by students. Risks to assessment validity include uncertainty in identifying struggling students and inability to intervene in a timely manner. The performance of ChatGPT on problems requiring increasing demands of cognitive reasoning warrants further research.

## Introduction

The ongoing development of artificial intelligence (AI) technology presents both medical educators and students with unprecedented opportunities to teach and learn. Whether it be facilitating the retrieval and summary of the medical literature, analyzing trends in student responses in real-time, or enhancing simulation exercises with virtual reality, AI technology has already begun to be embedded in education.^1,2^ Conversely, the exponential rate of progress of AI-powered tools can pose challenges to their thoughtful integration into routine teaching practices.^3,4^

In November 2022, ChatGPT (OpenAI, California, USA), a large language model (LLM), was released for public use. ChatGPT has a dialogue interface and can interpret complex free-text queries or prompts, responding to users with full sentences or paragraphs, mimicking human writing styles. The potential of ChatGPT to be harnessed in medical education is well described elsewhere.^5–7^ Within weeks of release, ChatGPT gained notoriety in the field of medical education for having performed at or near the passing threshold for the three components of the multiple-choice United States Medical Licensing Examination (USMLE).^8,9^

While licensing examinations in Canada and the United States are in the multiple-choice format, assessment throughout the medical curriculum includes other formats. Short answer assessments are a widely used form of student evaluation in medical education, and are critical in evaluating student knowledge, communication skills, and other essential competencies required for delivering compassionate care.^10,11^ The ability of ChatGPT to quickly and accurately apply known concepts in medicine to novel problems, including reflection prompts and exam questions, and to mimic human writing styles, introduces a potential threat to the validity of traditional forms of medical student assessment including short answer assessment. Although the magnitude of the threat is highly dependent on the environment and context of the administration of the assessment, technologies such as ChatGPT pose a particular threat to the unproctored, online assessments that have risen in popularity in post-COVID medical education curricula. There is a small body of research where ChatGPT has been applied to short answer problems in pathology,^12^ microbiology,^13^ and medical biochemistry,^14^ but these studies did not actively compare ChatGPT performance to human performance, nor did these studies focus on threats to assessment validity.

The purpose of this study was to determine the extent to which the use of ChatGPT threatens the scoring validity of short answer assessments in an undergraduate medical education program. By comparing the performance of ChatGPT and pre-clerkship medical students on short answer questions encountered on their regular assessments, we explored the extent to which responses are similar between groups for questions aimed at the six levels of Bloom's Taxonomy, a pedagogical framework that includes descriptions of six domains of cognitive problem solving with increasing levels of complexity (knowledge, comprehension, application, analysis, evaluation, synthesis).^15^

## Materials and Methods

### Context

McMaster University, located in Hamilton, Ontario, Canada, is home to the Michael G. DeGroote School of Medicine. Our undergraduate medical program is one of seventeen medical schools in Canada, and one of two Canadian medical schools that offer a three-year MD program rather than a traditional four-year program. During the first sixteen months of foundational study before beginning clinical rotations in clerkship, students are often referred to as “pre-clerkship students”.

Pre-clerkship students at our medical school write a concept application exercise (CAE) assessment approximately every 4 weeks covering the most recently covered curriculum content.^16^ CAEs feature four or five clinical scenarios each with multiple prompts and questions. Students provide one consolidated response in short answer format to each clinical scenario, attempting to respond to all of the information and questions provided. Students are given one hour to complete each CAE. Instructors in our medical program who work with students in small-group, problem-based learning tutorials for two to three months at a time (“tutors”) each grade their own students using a software platform that conceals the identity of each student until after all grades are submitted. Tutors are provided with a grading rubric for each problem. Each CAE scenario response is graded individually on a five-point scale:1 (Novice), 2 (Approaching Proficient), 3 (Proficient), 4 (Beyond Proficient), or 5 (Accomplished). A score of 3 is considered the minimum standard for level-appropriate understanding of the pathophysiology and management of the CAE problem.

### Data Collection

We collected a sample of forty CAE questions previously used for student assessment in our undergraduate program while ensuring each of the core units in the pre-clerkship curriculum was adequately represented. Questions from our CAE database with images, tables, or other supplementary figures were not included in the study as ChatGPT was not able to handle inputs of these formats at the time. Of the forty included questions, we randomly selected 30 questions (75%) to submit to ChatGPT (version 3.5, March 2023) and recorded the computer-generated responses. For the remaining ten questions (25%), we recorded past student responses which were previously evaluated at a level 3. The level of 3 out of a possible 5 marks was chosen as this represents the minimum standard for this assessment at our institution.

The questions were classified by Bloom's Taxonomy in order to determine if there exists a performance difference at different levels. For the thirty questions submitted to ChatGPT, there was an equal distribution of questions across each of the six levels of Bloom's cognitive domain. The ten questions selected with student responses were equally split between the lower (knowledge, comprehension, application) and higher (analysis, evaluation, synthesis) taxonomy levels.

Six experienced tutors from our undergraduate medical program each graded the sample of forty CAE problems. Tutors were provided with the grading template for each question and were asked to assign a score from 1 to 5 for each problem. The Fleiss interrater κ metric^17^ was used to measure consistency of evaluators to the problems. Optionally, each tutor was invited to provide free-text feedback to accompany their score for each problem.

The tutors were not involved in the selection of CAE problems or the collection of data from ChatGPT. Tutors were blinded to whether each response was provided by a student or ChatGPT. Tutors were unaware of the ratio of student-generated to computer-generated responses in the sample and were also unaware that all prior student answers were previously evaluated at a level 3 out of 5.

### Analysis

The scores assigned by each of the six tutors were averaged for each CAE question. The difference between the mean scores of students (n = 10) and ChatGPT (n = 30) was analyzed using a t-test for independent samples and one-way analysis of variance (ANOVA). Effect sizes were estimated with Cohen's d, where d values of 0.2 were considered small, 0.5 medium, and 0.8 large.^18,19^ Reliability was estimated with Cronbach alpha.^20^ Furthermore, mean score variation across the Bloom's Taxonomy levels in each of our student-generated and ChatGPT-generated response groups was measured with ANOVA.

In addition, aggregate first-year medical student performance from our most recent pre-clerkship student cohort (approximately two hundred students) was extracted for the same forty questions, representing evaluation from our entire pool of tutors in the undergraduate medical program grading under typical assessment circumstances. This data was used to compare scores assigned in this study by our six experienced tutors to historical scores. Moreover, we used this data to compare ChatGPT-generated responses to responses from our most recent pre-clerkship student cohort across Bloom's taxonomy.

All statistical analysis was performed using SPSS (v26, IBM, Redmond).

### Ethics

This study was exempted from ethics review by Hamilton Integrated Research Ethics Board.

## Results

**Figure 1.**
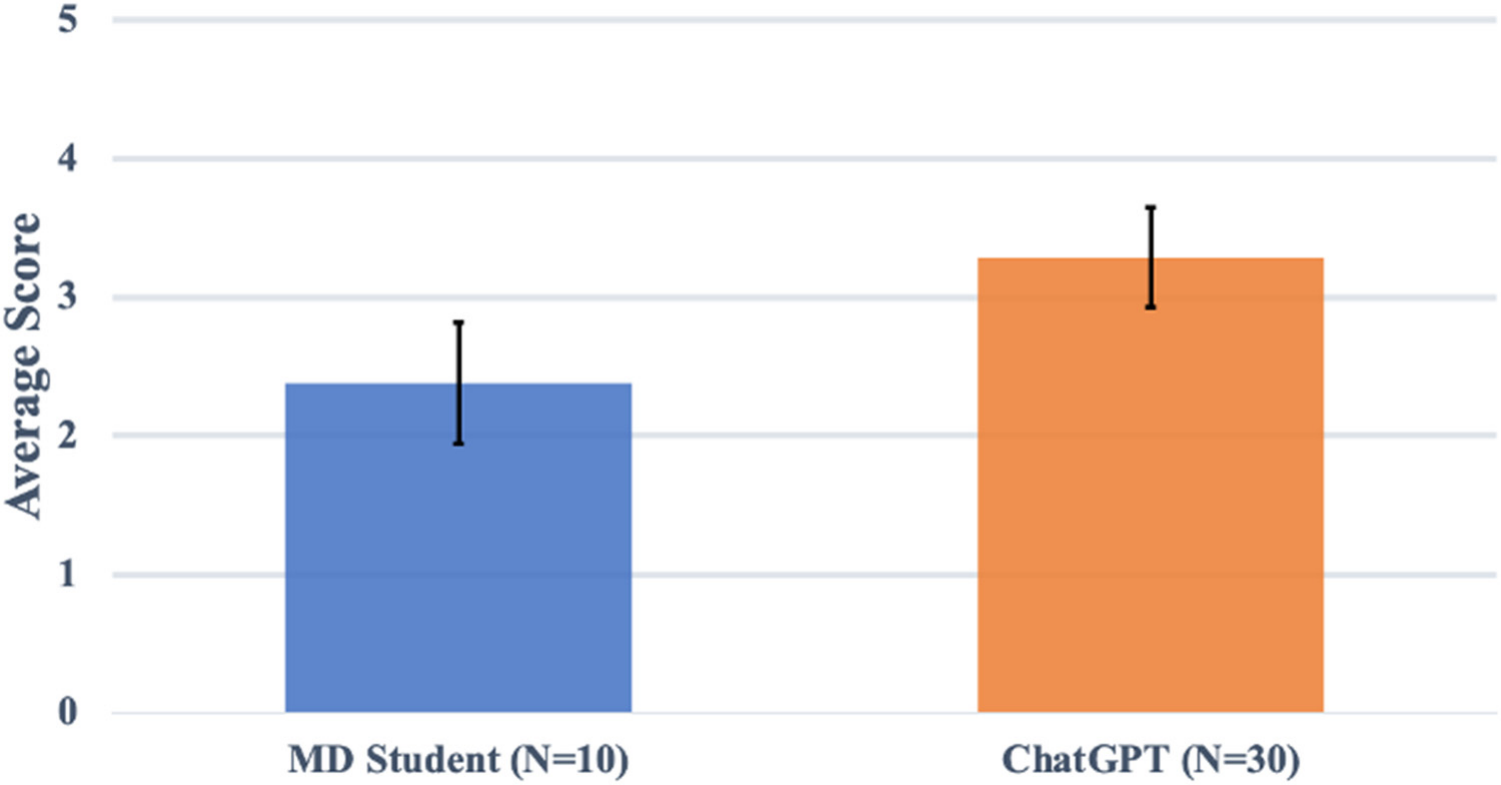
Mean performance of ChatGPT group and student group. The average score assigned by six tutors to ChatGPT-generated and student-generated short answer responses. The student-generated response sample consisted of previously assessed level 3 responses (from 1 to 5). In this limited sample, the mean score assigned to ChatGPT-generated responses was greater than the mean score assigned to student-generated responses at the passing standard (*P* = .008).

The average of the mean scores assigned by tutors to the ten student-generated responses (“minimally passing group”) was 2.38 (σ = 0.61, 95% CI 1.94-2.82, range 1.17-3.17) on a scale from 1 to 5 (see Figure 1). In comparison, the average of the mean scores assigned to the thirty ChatGPT-generated responses was 3.29 (σ = 0.96, 95% CI 2.93-3.65, range 1.67-4.67). One-way ANOVA with these groups showed a statistically significant difference between the mean scores assigned to the student-generated responses and the ChatGPT-generated responses (*P* = .008) and a moderate effect size (η^2^ = 0.169, 95% CI 0.012-0.368).

For the thirty questions that were posed to ChatGPT, our full historical student cohort achieved a mean score of 3.67. For the ten questions for which we retained student responses from the minimally passing group, the full historical student cohort had achieved a mean score of 3.55. Two-tailed t-tests showed significant outperformance by the full historical cohort of students when compared to both our current ChatGPT and the minimally passing group (see Table 1).

**Table 1.** Comparison of the two study groups to a recent pre-clerkship cohort.

	Average score from six tutors	Prior cohort average	Difference (experiment minus prior average)	95% CI of the difference	Two-tailed *P*-value	Cohen's d with Hedges correction
ChatGPT (n = 30) compared to prior cohort average	3.29	3.67	−0.38	(−0.70, −0.07)	.018	−0.44
Sample student responses (n = 10) compared to prior cohort average	2.38	3.55	−1.17	(−1.52, −0.82)	<.001	−2.20

Our most recent cohort received higher average scores when answering the same questions as our current study samples.

In measuring consistency between the six independent tutors, the Fleiss interrater κ score was found to be 0.137 (n = 30; 95% CI: 0.088, 0.186) for evaluations of the ChatGPT response group and 0.189 (n = 10; 95% CI: 0.083, 0.296) for the student response group. These values suggest a statistically significant but relatively low level of exact agreement across our tutor pool. We note that this score does not account for the ordinal nature of our five-point scoring system; that is, a scoring difference by one point between two tutors is accounted for as a disagreement.

The Cronbach alpha was found to be 0.890 for the ChatGPT-generated responses and 0.816 for the student-generated responses from our experiment. These values suggest strong levels of internal consistency and reliability for both groups. The mean inter-item correlation was measured as 0.588 (range: 0.356 to 0.799) for the ChatGPT-generated responses and 0.455 (range: −0.225 to 0.726) for the student-generated responses.

### Bloom's Taxonomy Domains

**Figure 2.**
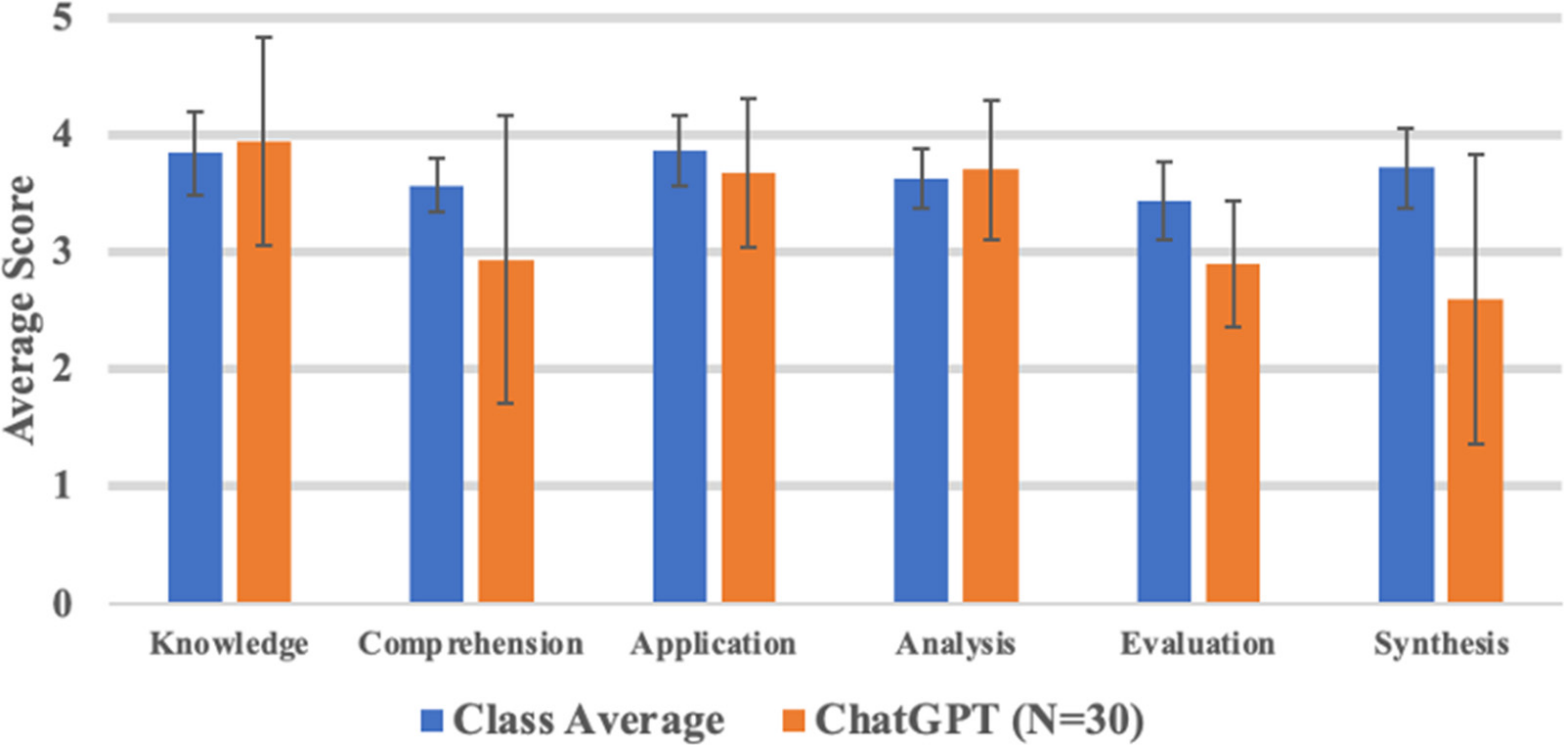
Performance of ChatGPT group and historical student cohort across Bloom's Taxonomy. Comparing the performance of ChatGPT across the six domains of Bloom's Taxonomy as measured by six tutors in this study to the average performance of the most recent pre-clerkship cohort as evaluated by our entire cohort of tutors on the same questions (n = 30; n = 5 per domain). No statistically significant differences exist in performance for any of the six domains.

The distribution of problem scores by domain of Bloom's Taxonomy for the ChatGPT group and the minimally passing group can be found in Table 2. One-way ANOVA across the six domains did not show a statistically significant difference when comparing the variation between domains or within domains for either ChatGPT (*P* = .146) or student responses (*P* = .174). Upon comparison to the performance of our most recent pre-clerkship student cohort by domain, no statistically significant differences or trends across the six domains was identified (see Figure 2).

**Table 2.** Score details of ChatGPT group and minimally passing student group across Bloom's taxonomy.

Bloom's Taxonomy Domain	Sample group	Number of problems	Mean score for group of problems	Minimum average score	Maximum average score	Standard deviation of average scores per group
Knowledge	ChatGPT	5	3.93	2.67	4.67	0.89
	Students	2	2.33	2.33	2.33	0
Comprehension	ChatGPT	5	2.93	1.83	4.50	1.23
	Students	2	2.67	2.33	3.00	0.47
Application	ChatGPT	5	3.67	3.00	4.33	0.63
	Students	1	1.17	–	–	–
Analysis	ChatGPT	5	3.70	3.17	4.67	0.59
	Students	1	3.17	–	–	–
Evaluation	ChatGPT	5	2.90	2.33	3.67	0.54
	Students	4	2.38	2.00	3.17	0.55
Synthesis	ChatGPT	5	2.60	1.67	4.67	1.23
	Students	0	–	–	–	–

Descriptive statistics from our experiment with questions posed to ChatGPT (n = 30) and students (n = 10) stratified by domains of Bloom's Taxonomy. Each of the questions per group was assigned a score by six tutors and the mean question for each score was analyzed.

### Free-text Tutor Comments

With six tutors grading forty problems, there were a total of two hundred and forty evaluations with corresponding opportunities for free-text tutor comments or feedback. Fifty-four comments were made in total. In twenty-five instances, tutors guessed whether the response was written by a student or ChatGPT. Of these instances, 64% of guesses were correct, and 36% incorrectly guessed that a ChatGPT-generated response was written by a human. In no instance did a tutor guess that a human-generated response was generated by ChatGPT. Twenty-two comments included specific feedback for the student or ChatGPT response, or justification for why a particular score was given. Eleven comments indicated a level of dissatisfaction with the rubric provided to grade the question including seven comments where it would have been useful to provide marking breakdown for each section of the case instead of assigning a global score to each problem.

## Discussion

In our experiment, ChatGPT-3.5 reliably achieved a passing grade on short-answer CAE problems delivered in our undergraduate medical program. This represents success on a different form of assessment compared to the multiple-choice format of USMLE, which ChatGPT has been shown to pass.^8,9^ ChatGPT performance was shown to be comparable to students who are performing at the minimum standard on these assessments, but slightly lower when compared to a historical student cohort. The level of performance was not found to vary across domains of Bloom's taxonomy.

Our primary objective in this study is to consider threats to validity of our short-answer CAE assessments in light of the release of ChatGPT. That is, we hope to ensure that new technology augments the learning experience for our students rather than diminishing meaningful assessment opportunities. Our findings, including the comments from tutors where ChatGPT-generated answers were mistaken for human answers on several occasions, prompt us to consider adjustments to our assessment strategy to protect the validity and value of our assessments. Possible adjustments include more strictly supervised assessment settings, alternative methods of assessment (eg, oral exams), using AI detection software, raising passing thresholds, or increasing the number of prompts with data in images (eg, medical imaging, pathology slides, physical exam findings) which ChatGPT is currently unable to process. However, it is likely that increasing question difficulty and including images has already been, or will soon be, rendered ineffective with the release of ChatGPT-4 and further iterations of the technology.

We found that the mean score assigned to ChatGPT responses was significantly greater than the score assigned to the minimum standard student group in this study. However, our findings affirmed that by selecting past student responses receiving a passing grade of 3 out of 5, we were selecting minimally passing student responses compared to a typical cohort of students, rather than selecting from the median performance standard of our students. That is, the result of this comparison suggests that ChatGPT outperforms minimally passing students. The primary purpose of CAE assessments is to identify students who may require additional support in reaching MD program objectives. With ChatGPT producing passing short-answer assessment responses that are not always distinguishable from student responses, the primary purpose of CAE assessments is brought into question as students requiring additional support may not be identified.

Our findings also suggest that our group of six tutors was more severe in their assignment of grades compared to our entire roster of tutors from our prior pre-clerkship student cohort. This may have been attributable to several factors. In actual practice, while our CAE problems are assigned scores of 1 to 5 based on a rubric, tutors are often more focused on providing written feedback to their students and deciding how to adjust their future problem-based learning sessions based on student understanding, rather than focusing on a single number to summarize performance. This is in contrast to the current study where the primary objective of scoring was to assign a value for comparison purposes and the responses were not generated by students who they were personally teaching for an extended period of time. Moreover, the tutors selected for this study were very experienced in grading CAEs which may have contributed to higher grading expectations than the average tutor from our roster. While interrater reliability (kappa) scores were fairly low in this study, this evaluation metric is limited in our context of ordinal data.

While the grading environment in this experiment was not reflective of the actual learning environment, the results remain useful to address our study objectives. Repeating the experiment by combining ChatGPT-generated responses with higher quality student responses (eg, scoring 4 or 5 out of 5) may be a useful technique for future planned qualitative research studies, for example, to identify themes and patterns in the way that high-achieving scores are produced by students compared to ChatGPT. We note that further software updates and advanced models may continue to surpass ChatGPT's current level of performance, including ChatGPT-4 released in April 2023 after the conclusion of this experiment which is able to process images. Preliminary experiments have suggested that ChatGPT-4 outperforms ChatGPT-3.5 on several well-known standardized exams such as the LSAT and GRE for admissions to graduate programs and the Medical Knowledge Self-Assessment Program.^21^ Furthermore, the ability of this new iteration to interpret images has led to further outperformance on many exams.

Our limited sample did not reveal a statistically significant trend in the quality of ChatGPT responses in association with question complexity, which in our study is measured by domain of Bloom's Taxonomy. While it might be reasonable to expect ChatGPT responses to diminish in quality with higher Bloom's domains, it is also important to consider that its training data would also have included texts, topics, and problems covering all domains, and that the machine learning models are able to learn patterns and associations across its entire training corpus. This lack of trend in ChatGPT response quality with question difficulty or complexity is in contrast with one of the USMLE studies^9^ and warrants further focused research. If the quality or accuracy of ChatGPT responses were to decrease in higher Bloom's domains, shifting student assessment more heavily to these domains may also secure the validity of these assessments.

This study examined the impact of the use of an AI tool on existing assessment practices. The extent to which medical students and residents perceive and engage with such tools is also being measured in the literature. The attitudes of learners toward AI in medicine are mixed, although it has been suggested that training in AI is associated with more positive attitudes toward its use.^22^ Such training can include how to interpret the output of models and the consideration of bias in their underlying machine-learning models.^23^ Despite the very limited presence of autonomous AI in medical practice at this time, training in AI is likely to benefit medical learners given that clinical groups are beginning to actively explore how to further harness AI in their practices,^24^ especially in the context of the known current knowledge gap for medical learners in emerging technologies.^25^ The increasing capabilities of AI models and the quality of AI training in medical education may also influence the career goals and preferred residency disciplines of medical students, similarly to what has been preliminarily observed in diagnostic radiology.^25–27^

ChatGPT may become an instrumental teaching tool to help students master the undergraduate medical curriculum, although the degree to which it can and should be incorporated into medical education has understandably become a controversial subject. Our group supports the thoughtful and ethical incorporation of artificial intelligence in medical education^28^ and are actively exploring ways that these tools might be used to support underperforming students. Moreover, calls to incorporate knowledge of artificial intelligence in medical education, including appropriate use, continue to build^29,30^ and there is now gradual incorporation into Canadian medical training.^31,32^

## Conclusions

The ability of ChatGPT to meet the standard on our institution's short answer assessment problems poses a threat to the validity of our assessments, especially given our assessor comments that they were sometimes convinced that a ChatGPT-generated response was produced by a student. Its ability to respond to problems across the domains of Bloom's Taxonomy warrants further research. Our most recent pre-clerkship cohort outperforms the average performance of ChatGPT but there is a risk of underperforming students not being identified in a timely manner if the technology is wrongfully applied by students on assessments.
